# A contemporary look at allergic conjunctivitis

**DOI:** 10.1186/s13223-020-0403-9

**Published:** 2020-01-21

**Authors:** Pascale Dupuis, C. Lisa Prokopich, Alexander Hynes, Harold Kim

**Affiliations:** 1 Division of Clinical Immunology and Allergy, Department of Medicine, St. Joseph’s Hospital, Western University, Room B3-102, 268 Grosvenor Street, London, ON N6A 4V2 Canada; 2 School of Optometry & Vision Science, University of Waterloo, 200 Columbia St W., Waterloo, ON N2L 3G1 Canada; 3 Kitchener, Canada; 4 Division of Clinical Immunology and Allergy, Department of Medicine, McMaster University Health Sciences Centre, 1280 Main St. W., Hamilton, ON L8S 4K1 Canada

**Keywords:** Allergic conjunctivitis, Allergic eye disease, Ocular allergy, Allergic conjunctivitis diagnosis, Allergic conjunctivitis treatment algorithm, Interprofessional management

## Abstract

Allergic eye disease is common, yet often overlooked in North America. In the U.S., up to 40% of the population is deemed to be affected and this number is growing. Symptoms and signs of ocular allergy can lead to decreased productivity and negatively impact quality of life (QoL). Various treatment options exist to achieve symptom control. For allergic conjunctivitis, ophthalmic agents include antihistamines, mast cell stabilizers, dual-activity agents, nonsteroidal anti-inflammatory drugs (NSAIDs), steroids and some off-label treatments. Immunotherapy is recommended as a therapeutic option. This review provides a summary of the forms of ocular allergies, with a focus on symptoms and signs, impact on QoL, physical examination, diagnosis and therapeutic options of allergic conjunctivitis. Through multidisciplinary collaborations, a simplified algorithm for the treatment of allergic conjunctivitis is proposed for Canadian clinical practice.

## Background

**Fig. 1:**
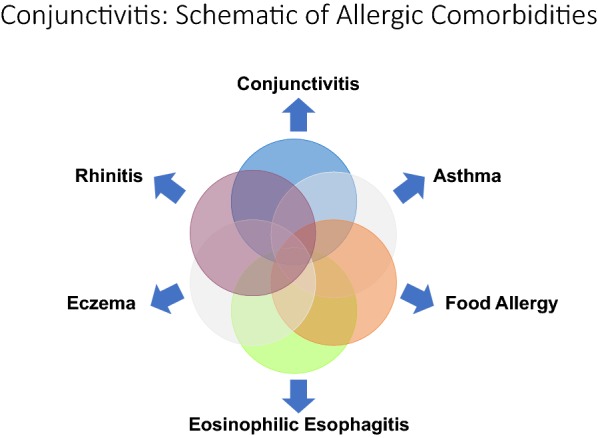
Conjunctivitis: Schematic of allergic comorbidities [5, 6]

Allergic eye disease is common, affecting approximately 40% of the North American population and increasing in prevalence [1–3]. Most patients suffer from concomitant allergic rhinitis, although 6% have isolated ocular symptoms [2]. Up to 44% of children and 20% of adults with asthma have symptoms suggestive of allergic conjunctivitis (AC) [4]. There are also established links between allergic rhinoconjunctivitis and other atopic conditions including asthma, eczema, food allergy and eosinophilic esophagitis (Fig. 1) [5, 6]. This highlights the importance of obtaining a targeted ocular history during patient evaluation to appropriately assess ocular involvement.

Traditionally, less attention has been paid to this entity compared to other allergic diseases such as allergic rhinitis. Due to a lack of awareness from both patients and health care professionals, many continue to be underdiagnosed and undertreated [7]. Patients often self-medicate and/or fail to seek help for their ocular symptoms, leading to poor symptom control and decreased quality of life and productivity [1, 8]. Diagnosis and treatment is essential to ensure symptom relief and to prevent complications that can arise from untreated disease.

This article provides an overview of AC, the most common form of allergic eye disease, by discussing the pathophysiology, epidemiology, characteristics of the disease, diagnosis, management options and impact of quality of life. A simplified algorithm outlining treatment of AC is included to provide step-by-step guidance to health care professionals. This review also emphasizes the value of interprofessional collaboration to enhance patient care.

## Eye anatomy and immunologic function

**Fig. 2:**
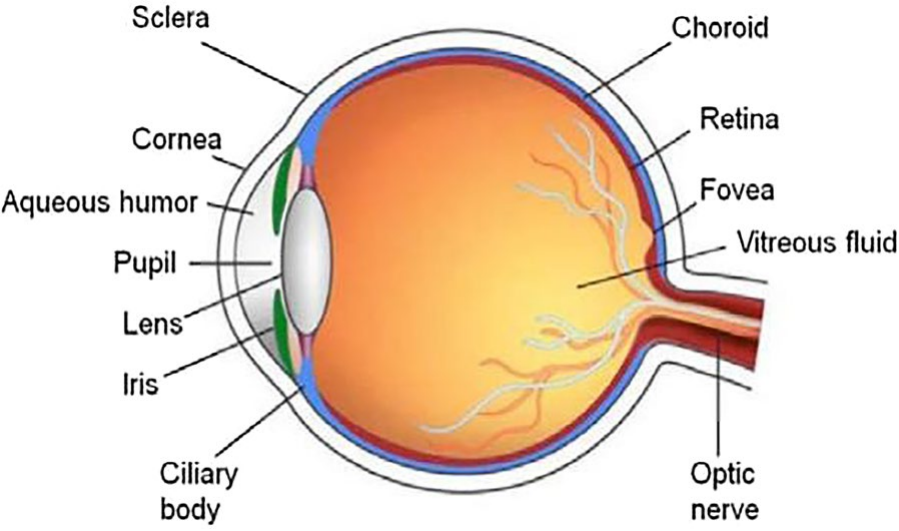
Cross-sectional anatomy of the eye [12]

The eye is intricate and each part plays a specific immunologic role (Fig. 2). The eyelids act as a barrier to insult, including to allergens. The lacrimal functional unit produces the tear film, which provides lubrication and protection [9]. Inflammatory conditions such as AC can alter the composition and volume of tear production [10]. The conjunctiva and cornea are the most external layer that come in contact with environmental allergens. The normal conjunctiva does not contain mast cells; they reside just below, in the superficial portion of the substantia propria, along with the other inflammatory cells [10]. In AC, there in an increase in conjunctival mast cells and eosinophils. The cornea is avascular and is seldomly involved in AC, although alterations of the corneal cells may lead to blurry vision and changes in visual acuity. The sclera sits below the conjunctiva. Its major constituent is collagen and it is the primary ocular site involved in diseases affecting connective tissues (e.g., rheumatic disorders) [11]. The uvea is highly vascular and produces aqueous humor. Inflammation of the uvea (uveitis) is predominantly associated with infectious and autoimmune conditions. The retina and optic nerve relay the information from the surrounding world to the visual cortex and can be affected in systemic diseases such as vasculitides.

## Pathophysiology

The ocular mucosa has a large surface area. It is therefore one of the most accessible sites allowing direct antigen deposition, leading to the initiation of the allergic cascade.

AC is the only ocular disease to involve solely a type I allergic reaction [13]. In sensitized individuals, Th2 cells release pro-inflammatory cytokines (IL-3, IL-4, IL-5, IL-13) that stimulate immunoglobulin E (IgE) production by the B cells [14]. The IgE become membrane-bound to mast cells and subsequent cross-linking by their respective allergens triggers mast cell degranulation and release of preformed (histamine, tryptase) and newly formed mediators (leukotrienes, prostaglandins) [10, 14].

The early phase of the allergic cascade begins within seconds to minutes after exposure and clinically lasts 20–30 minutes [13]. During the early phase, mast cell release of mediators cause symptoms such as pruritus, tearing, redness, conjunctival injection, chemosis and a papillary reaction [15]. The late phase begins a few hours later and is characterized by epithelial infiltration of inflammatory cells like neutrophils, lymphocytes, basophils and eosinophils, which lead to continued inflammation, persistent symptoms and increased likelihood of tissue damage [13, 14]. As the reaction progresses, hypersecretion of tears increases drainage through the lacrimal ducts carrying allergens directly into the nasal passage [15].

## Types of allergic conjunctivitis and other allergic eye diseases

**Fig. 3:**
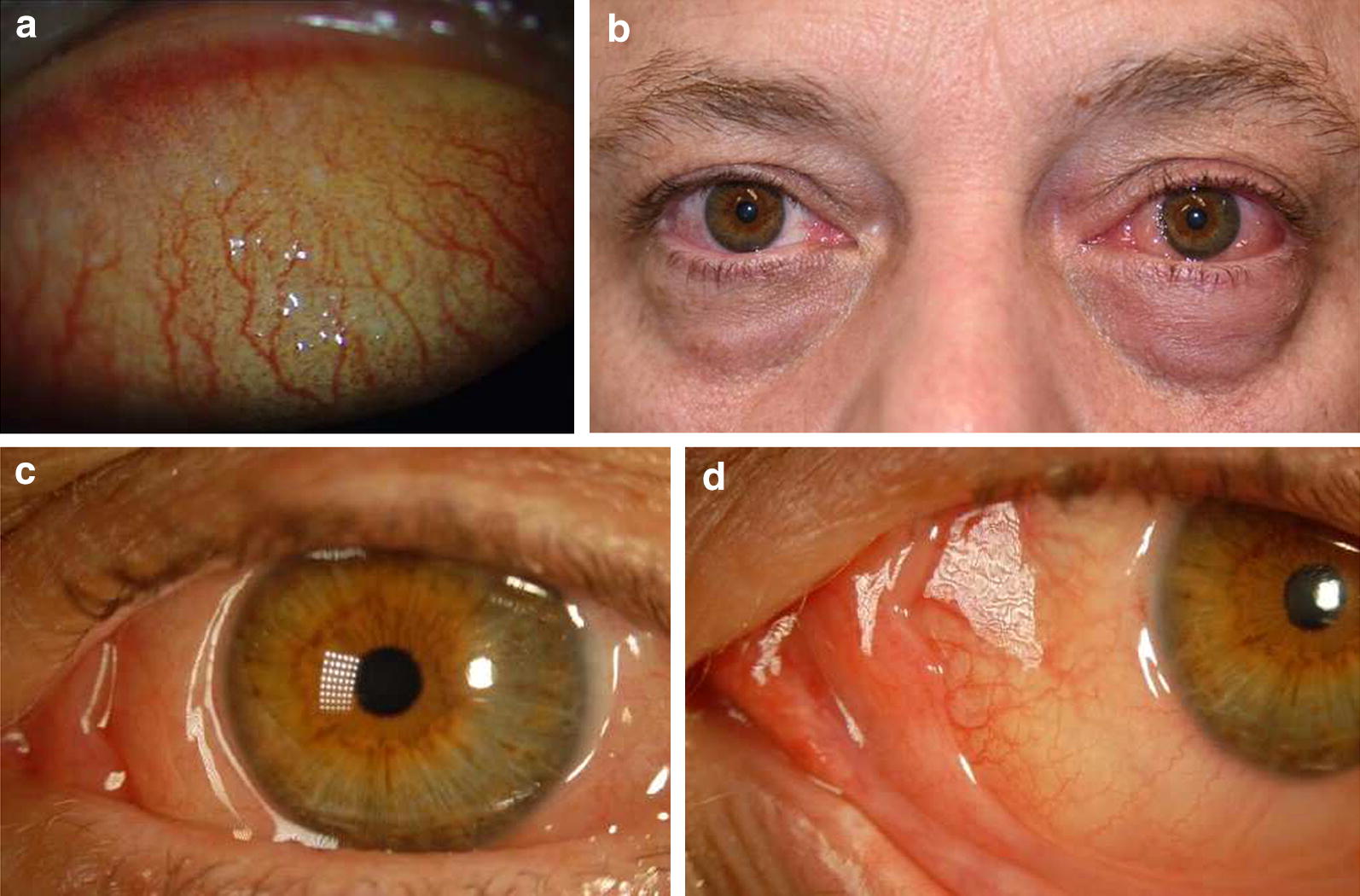
Ocular signs of (**a**) chronic perennial allergic conjunctivitis (**b**–**d**) acute seasonal allergic conjunctivitis. Reproduced with permission [15]

AC is further categorized as seasonal and perennial, the former being more common (Fig. 3a–d) [10, 16]. The difference between the two conditions is simply the periodicity or chronicity of the symptoms, which is dictated by the type of allergen patients are sensitized to [7].

Seasonal symptoms are triggered by transitory allergens such as tree or grass pollens. Perennial symptoms are caused by indoor allergens such as house dust mites, animal dander, mold spores, cockroach or rodents [7]. The smaller allergens have the potential to cause more symptoms, as they can more easily become volatile. For example, cat, dog and rodent dander is smaller and tends to cause more eye symptoms than house dust mites or cockroach antigen, which cannot remain airborne for more than a few minutes after disturbance [17]. Many patients are polysensitized and experience perennial symptoms with seasonal exacerbations.

**Fig. 4:**
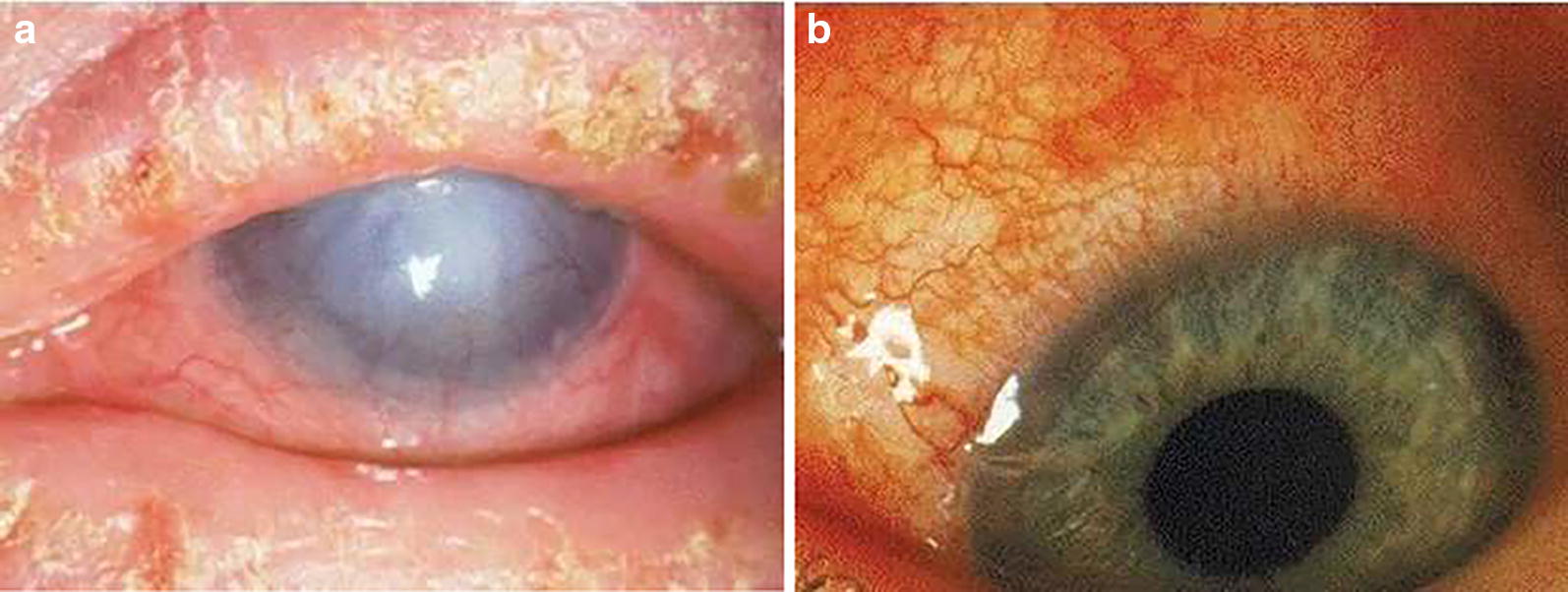
Ocular signs of (**a**) atopic keratoconjunctivitis and (**b**) vernal keratoconjunctivitis. Reproduced with permission [15]

Perennial and seasonal AC are not only the most common but are also the mildest forms of ocular allergic disease. Atopic keratoconjunctivitis (AKC, Fig. 4a) and vernal keratoconjunctivitis (VKC, Fig. 4b) lead to epithelium remodeling and in rare cases vision loss [18, 19]. Giant papillary conjunctivitis (GPC) or more appropriately termed contact lens papillary conjunctivitis (CLPC), is traditionally included in the group of ocular allergic diseases, although it has been found to be the result of nonimmune tissue damage from repetitive micro-trauma, usually in contact lens wearers [20, 21]. Each condition is summarized in Table 1.

**Table 1:** Other ocular allergic subtypes and their main characteristics

Ocular allergy subtype	Demographics and/or associations	Primary symptoms and signs
Atopic keratoconjunctivitis (AKC) [10, 15, 18]	Male predominance, 30–50 years of age Perennial, with potential exacerbation in the winter months Association with atopic dermatitis of the eyelids	Severe ocular itching (ocular surface, eyelids) Tearing, burning, photophobia, mucous discharge Significant hyperemia and edema of the conjunctiva (chronic inflammation) Corneal scarring, neovascularization Trantas’ dots Large cobblestone papillae on superior tarsus and/or limbus (chronic inflammation)
Vernal keratoconjunctivitis (VKC) [10, 15, 18]	Male predominance, 3–25 years of age Associated with atopy in 50%	Severe ocular itching Photophobia, tearing mucous discharge Trantas’ dots (limbal form) Large cobblestone papillae on superior tarsus and/or limbus (chronic inflammation) Corneal ulcer (shield) may form in severe cases

## Characteristics of allergic conjunctivitis—impact and diagnosis

### Importance of appropriate professional care

Due to its non life-threatening nature, AC typically receives less attention than other chronic conditions with higher morbidity or higher mortality rate. Despite the prevalence of the disease, up to a third of patients with the disease continue to be underdiagnosed and undertreated [22].

Patients often self-medicate with purchased over-the-counter (OTC) medications and fail to seek help even when those therapies are ineffective [22, 23]. In one study, 56% of patients diagnosed with AC started with self-treatment measures as first action. Washing the eyes with water or saline were the most commonly chosen therapies [23]. Many OTC drugs have limited efficacy for AC (e.g., topical vasoconstrictors) and can lead to undesirable side effects (e.g., rebound vasodilation from topical vasoconstrictors; mucosal dryness or drowsiness from oral antihistamines).

Furthermore, the use and overuse of OTC products can lead to adverse effects in various ocular issues. Concern exists over the preservatives found in OTC eye drops, which can increase ocular toxicity and exacerbate ocular surface symptoms. As an example, benzalkonium chloride, a common preservative found in 70% of OTC eye drops, is known to cause corneal epithelial cell damage in predisposed individuals or with prolonged exposure [24, 25].

Certain diagnostic considerations and treatments require specific care and follow-up by an optometrist or ophthalmologist. A detailed case history and direct physical examination of the eye and adnexa including evaluation by slit-lamp biomicroscopy are paramount in the evaluation of moderate and severe AC, both to confirm the diagnosis and rule-out other ocular diseases that may require different treatment considerations. Ophthalmic steroid drops are effective for the treatment of AC, although prolonged therapy with steroids requires close supervision and frequent eye examinations by an optometrist or ophthalmologist due to increased risk of elevated intra-ocular pressure, development of cataract and central serous chorioretinopathy as well as other less likely concerns such as ptosis, mydriasis and eyelid skin thinning in the case of skin applications [26–28].

When maximal medical therapy insufficiently relieves the symptoms, other treatment considerations such as immunotherapy (IT) by an allergist can be explored. In one study looking at patients diagnosed with AC by ophthalmologists, only 37% had received an allergy evaluation [23]. Referral to the eye care specialist or allergist is discussed below.

### Symptoms and signs

The most common feature of AC is pruritus, which can range from mild to severely debilitating [18]. Rarely, it may be described as painful. Other symptoms include tearing, redness, foreign body sensation, mucous discharge and eyelid swelling [11, 16]. Symptoms are typically bilateral and associated with rhinitis [16]. Blurred vision and photophobia can occur in severe cases [29]. Other symptoms including patchy redness and flaking of the eyelid skin and contact lens intolerance are helpful.

The patient may not be symptomatic at the time of the visit, so probing about time of year when symptoms are most severe is important. Those symptoms are not specific to AC and could be the result of various nonallergic conditions, hence the importance of obtaining an accurate patient history.

Patients with allergic eye disease frequently suffer from other allergic comorbidities (Fig. 1). It is important to enquire about the symptoms and signs of other common allergic processes during patient evaluation to obtain a more complete picture of their illness.

A thorough history of current and previous medications used along with an evaluation of relative symptom relief helps confirm the diagnosis. In children, a diagnosis of attention deficit hyperactivity disorder (ADHD) has been associated with a higher likelihood of suffering from allergic conjunctivitis [30]. Evaluation of family history of atopic co-morbidities such as allergic rhinitis, atopic dermatitis or asthma increase the likelihood of an allergic disorder. Exposure to highly allergenic elements in the environment (e.g., pets, pest, molds, pollens) with a timeline of symptom exacerbations helps determine triggers. Exposure to other ocular triggers should be explored, for example smoking, occupational exposures, personal protective equipment used if any or long-term use of contact lenses. Table 2 summarizes key points to obtain on history.

**Table 2:** Components of a complete history for suspected ocular allergy [7, 10, 15]

Category	Question for patients
Ocular symptoms	What are your symptoms? How severe are they? Are your eyes itchy? Do they burn? sting? Are they painful? Is there discharge from your eyes? If so, is it watery or mucoid? Does it feel like there is a foreign body in your eyes? Do you rub your eyes? Are your eyes dry? When did your symptoms start? What is your worst season, if any? Have you had any previous episodes? Are your symptoms in one eye or both? Are there any exacerbating or relieving factors? Is your vision affected? Are you sensitive to lights? Do you wear contact lenses? Are they comfortable? Is there any history of trauma to your eyes?
Health history	Is there associated atopy? Or a family history of atopy? Is there a diagnosis of ADHD? Are you on any medications? Are there any other past medical and surgical concerns (tonsillectomy, sinus surgery)?
Exposures/Environment	Do you live with pets? Is the home carpeted? Forced-air heating? Air conditioning? Humidity level? Is there exposure to smoke (first- or second-hand)? Have there been any new exposures (e.g., new pet, renovations, new personal or home hygiene products)? Are there any potential occupational exposures? Infectious contacts (possibility of infectious cause of red eye)?
Treatment	Have OTC topical products been used? If so, which product(s)? Have OTC oral agents been used? If so, which product(s)? Have prescription medications, including immunotherapy, been tried? How often were the therapies used and for how long? Has there been any relief of symptoms?
Quality of Life	Are the symptoms interfering with school/work, activities of daily living or sleep? Has school/work been missed due to symptoms?

It should be noted that patients suffering from dry eye disease may also report ocular itching [20]. The dominant symptoms of dry eye disease include dryness, discomfort, burning, stinging and foreign body sensation [20]. The two conditions share some similar clinical features of the ocular surface, and differentiating between the two can be challenging. Moreover, the two conditions are not mutually exclusive and there is a growing body of evidence suggesting AC may be a risk factor for dry eye disease [31].

### Impact on quality of life

Interference with activities and effect on overall quality of life should be explored. Symptoms of allergies have a substantial impact on quality of life, especially when at their peak [23, 32, 33]. In a large population-based survey, red and itchy eyes were found to be the second most bothersome symptom of allergies, following nasal congestion. However, there was no statistical difference between the distress caused by nasal and eye symptoms [34]. The findings of negative emotions (irritability, frustration, anger, embarrassment), decreased productivity, decreased concentration, fatigue and absenteeism from work are consistent [23, 33, 34].

The economic burden of the disease is also increasingly recognized. This includes direct costs such as medications and visits to health care providers, as well as indirect costs such as missed days of work and decreased productivity while at work [35]. Although no data are available related to the cost of AC specifically, the direct annual cost of allergic rhinoconjunctivitis is estimated at $2–5 billion in the United States [36].

### Differential diagnosis

Since many ocular conditions may mimic the symptoms of AC, maintaining a broad differential is essential. The more severe forms of ocular allergy (AKC, VKC, atopic dermatitis), contact-lens associated papillary conjunctivitis, infectious causes, dry eye disease types, ocular toxicity from preservatives, ocular rosacea as well as blepharitis must all be considered [10, 16, 29].

### Physical examination

Assessment of symptomatic patients must include gross visual examination. We recommend slit-lamp biomicroscopy of the periocular and ocular tissues, including high magnification assessment of the cornea and limbus, in moderate and severe disease, although all patients may benefit from a detailed examination regardless of the severity of disease.

Biomicroscopy involves assessment of the lids and lashes, lid margins and Meibomian glands, tear film (including discharge), bulbar and palpebral conjunctiva (conjunctiva overlying the sclera and underlying the eyelids, respectively), and cornea [7, 15]. When available, fluorescein ocular surface staining under cobalt blue light examination can help identify epithelial disruption and highlight conjunctival irregularities such as papillae [11, 29].

A common technique for examination of the affected tissues includes eversion of both upper and lower eyelids with the help of a cotton-swab. For examination of the lower eyelid, the patient is asked to look up and the eyelid is pulled down and observed under the slit-lamp. For examination of the upper eyelid, the cotton-swab is applied on the upper lid at the superior margin of the tarsus while the patient is asked the look down. The eyelashes are gently grasped and the eyelid is pulled out and flipped up over the cotton-swab. For examination of the lower eyelid, the patient is asked to look up and the eyelid pulled down over the cotton-swab. This is helpful to evaluate for injection and papillary changes and to differentiate other findings [11].

Patients with AC may have unremarkable physical findings on gross observation, especially if they are seen outside of exacerbations. The eyelids may be hyperemic and edematous and this can be more marked in the lower eyelid due to gravity. An allergic ‘shiner’, a bluish discoloration below the eyes, may be present in acute disease, and is as a result of venous congestion [7]. During acute or chronic exposures, watery discharge may be noted, but mucous discharge may also be visualized in the tear film [28]. Otherwise, bilateral conjunctival injection is the most obvious general finding. Chemosis, swelling of the conjunctiva, can be moderate to severe in acute episodes and may be somewhat disproportionately more prominent than the degree of redness found on examination [11, 18]. When severe, the conjunctiva appears gelatinous and may be thickened to the point that the cornea appears to be recessed. This can cause ocular complications as blinking may fail to protect the corneal surface. Otherwise, corneal involvement is rare, but it is of critical importance when it is identified as it may differentiate more chronic allergic disease types and alter treatment recommendations. Table 3 lists the ocular examination findings of AC.

**Table 3:** Ocular examination findings of allergic conjunctivitis [15]

Ocular structure	Associated findings
Lids/lashes	Lid hyperemia/edema Ptosis Allergic ‘shiner’
Tears	Watery, occasionally mucoid
Bulbar conjunctiva	Superficial injection Chemosis (if severe, may cause ‘hour glass’ appearance)
Palpebral conjunctiva	Injection Inferior or superior papillae (on lid eversion)
Cornea	Clear

Slit-lamp biomicroscopic examination is used to confirm the above findings, to exclude complications from other forms of ocular allergies and to rule out other diagnoses. Signs such as giant papillae, corneal infiltration, pannus, neovascularization and ulceration indicates an alternate diagnosis to AC. Table 4 lists the ocular examination findings of common ocular comorbidities.

**Table 4:** Ocular examination findings of common ocular comorbidities

Related ocular disease	Ocular signs
Allergic	
Atopic keratoconjunctivitis	Eyelid atopic dermatitis often present Conjunctival injection and chemosis Conjunctival scarring Giant papillae may be present Infiltration of the limbus (region where the cornea meets the sclera) and cornea [15]
Vernal keratoconjunctivitis	Tearing, profuse mucous discharge [28] Bulbar conjunctival injection Large papillae of superior palpebral conjunctiva, ‘cobblestone-like’ Corneal plaque/shield ulcer Trantas’ dots (infiltrates at the juncture of the cornea and the sclera) Corneal neovascularization and scarring
Atopic dermatitis	Periocular scaly, dry skin Eyelid thickening Lash loss Papillary hypertrophy of palpebral conjunctiva May be accompanied by conjunctival injection, watery/mucoid discharge [28]
Demodex-associated conjunctivitis (hypersensitivity to lid mites)	Heavy lash debris (lash collarettes) Bulbar conjunctival injection, may show papillae Eyelid hyperemia
Others	
Contact-lens associated papillary conjunctivitis, CLPC (often termed Giant papillary conjunctivitis, GPC)	Mucoid discharge Excessive movement of contact lenses Papillary hypertrophy of superior palpebra conjunctiva; if severe: lid swelling, ptosis [28] Clear cornea
Anterior blepharitis (staphylococcal, seborrheic) Posterior blepharitis (Meibomian gland dysfunction, ocular rosacea)	Lash debris, lid hypertrophy/hyperemia [15] Conjunctival injection and staining (lissamine green) Corneal staining (fluorescein) Evaporative dry eye disease
Dry eye disease (aqueous deficiency, evaporative)	Inadequate tear volume (low tear meniscus) (aqueous deficiency) Lash debris, lid hypertrophy/hyperemia, Meibomian gland dysfunction, ocular rosacea (poor tear film stability; evaporative dry eye) Conjunctival injection [15] and staining (lissamine green) Conjunctival chalasis (redundancy of the conjunctiva from loss of adherence to the sclera) Corneal staining (fluorescein)
Ocular toxicity (due to ophthalmic agents, usually preservatives)	Conjunctival injection Corneal staining
Others: e.g. superior limbic keratoconjunctivitis, floppy eyelid syndrome, etc.	Chronic symptoms and signs, some of which may overlap with AC

Briefly, the remainder of the physical examination should include assessment of the nasal passages for rhinorrhea and/or congestion, oropharynx examination, palpation of cervical lymphadenopathy, skin examination for concomitant atopic dermatitis and lung evaluation for signs of asthma.

### Supportive diagnostic testing

An allergy assessment should be sought when considering the diagnosis of AC. The standard allergy evaluation is undertaken by aeroallergen skin prick testing on the forearm, which has high sensitivity [37]. When the resulting wheal is at least 3 mm larger than the negative control, the result is considered positive [37]. Rarely, systemic reactions have been reported after skin prick testing [38]. If skin testing is indicated but not advised (e.g., the patient is taking medications with antihistaminic properties that cannot be discontinued), if the results are ambiguous (e.g., presence of dermatographism) or simply to complement the results of previous SPT, serum specific IgE measurements for the aeroallergens can be considered.

The conjunctival allergen challenge involves instillation of an allergen on the ocular surface with subsequent evaluation of the local response. A control solution is instilled in the other eye [39]. This is predominantly done in research settings to test novel treatments or to compare existing therapies. In the clinical setting, the conjunctival allergen challenge is underused yet is particularly helpful to identify local allergies in patients who have symptoms suggestive of AC but who have negative or discordant skin prick testing and serum specific IgE [29]. The challenge is also useful to assess the relationship between symptoms and exposure in polysensitized patients and to assess response to therapy after it has been initiated [29, 39].

### Management

Health care providers have access to a growing selection of treatments available for AC. The aim is to stop or minimize the inflammatory cascade associated with the allergic response in order to provide relief of symptoms and to prevent complications associated with prolonged inflammation. Although the initial treatment is often empiric, selecting therapies tailored to the patient’s specific symptoms may enhance response to treatment and improve treatment adherence. Immunotherapy is the only disease-modifying treatment available for allergic diseases including AC and may provide lasting benefit after desensitization is completed [7]. Table 5 provides a summary of the ophthalmic agents available in Canada and the U.S. for the treatment of AC.

**Table 5:** Ophthalmic agents available in Canada and the U.S. for the treatment of allergic conjunctivitis [15]

Agents (brand name)	Availability^a^	OTC/Rx	Year of market availability^b^	Age indication^c^	Dosing schedule
Topical ocular vasoconstrictors					
Naphazoline hydrochloride	Both	OTC	Established		Maximum QID, short term
Tetrahydrozoline hydrochloride	Both	OTC	Established		Maximum QID, short term
Ocular antihistamines					
Antazoline (only found in combination)	Both	OTC	Before 1980	N/A^d^	QID
Pheniramine (only found in combination)	Both	OTC	Before 1980	N/A^d^	QID
Emedastine 0.05% (Emadine^®^) [104]	U.S. only	Rx	1998	˃ 3 years	QID
Mast-cell stabilizers					
Lodoxamide 0.1% (Alomide^®^) [105]	Both	Rx	1992	≥ 4 years	QID
Cromolyn sodium 2%[106, 107]	Both	OTC/Rx	1993	≥ 5 years	QID
Dual-activity agents					
Olopatadine 0.1% (Patanol^®^)[108]	Both	Rx	1998	≥ 3 years	BID
Olopatadine 0.2% (Pataday^®^) [109]	Both	Rx	2011	≥ 16 years	Daily
Olopatadine 0.7% (Pazeo^®^) [110]	Both	Rx	2017	≥ 2 years	Daily
Ketotifen 0.025% (Zaditor^®^) [111, 112]	Both	Rx (OTC in U.S)	2000	˃ 3 years	BID to TID
Ketotifen 0.025% preservative free	U.S. only	OTC	2000	˃ 3 years	BID to TID
Bepotastine besilate 1.5% (Bepreve^®^)[113]	Both	Rx	2017	≥ 3 years	BID
Alcaftadine 0.25% (Lastacaft^®^) [114]	U.S. only	Rx	2014 (U.S. only)	≥ 3 years	Daily
Epinastine 0.05% (Elestat^®^) [115]	U.S. only	Rx	2004 (U.S. only)	≥ 3 years	BID
Azelastine 0.05% (Optivar^®^) [116]	U.S. only	Rx	2009 (U.S. only)	˃ 3 years	BID
Ophthalmic steroids (only some most commonly used in ocular allergy)					
Fluorometholone acetate 0.1% (FML^®^)^e^ [117]	Both	Rx	1972	˃ 2 years	BID^f^
Prednisolone acetate 1.0% (Pred Forte^®^)^e^ [118]	Both	Rx	1974	All ages	BID^f^
Loteprednol etabonate 0.2% (Alrex^®^)[119]	Both	Rx	2009	≥ 18 years	QID^f^
Loteprednol etabonate 0.5% (Lotemax^®^ (or Lotemax gel^®^))^e^ [120]	Both	Rx	2009	≥ 18 years	QID^f^
NSAIDs					
Diclofenac 0.1% (Voltaren Ophtha^®^)^e^ [121]	Both	Rx	1991	≥ 18 years	QID
Ketorolac 0.4% (Acular LS^®^) and 0.5% (Acular^®^)^e^ [75]	Both	Rx	1992 (0.5%) 2004 (0.4%)	≥ 18 years	QID
Nepafenac 0.1% (Nevanac^®^)^e^ [122]	Both	Rx	2008	≥ 18 years	TID
Bromfenac 0.7% (Prolensa^®^)^e^ [123]	Both	Rx	2015	≥ 18 years	Daily

*BID* twice daily, *N/A* not available, *NSAIDs* nonsteroidal anti-inflammatory drugs, *OTC* over-the-counter, *QID* four times a day, *Rx* prescription, *TID* three times a day

^a^“Both” indicates the agent is available in both Canada and U.S.

^b^Unless otherwise stated, the year of market availability in Canada

^c^For agents that are available in both Canada and U.S., the age indication is based on the Canadian product monograph

^d^Information not available

^e^Off-label use only in Canada; short term

^f^Or according to the severity of symptoms/inflammation

#### Allergen avoidance

Allergen avoidance is part of routine recommendations; however, not only is clinical benefit unclear but also true avoidance can be difficult to achieve. The following recommendations may be helpful in reducing allergen exposure.

Pollen and outdoor mold exposure can be reduced by keeping windows closed, using screen filters, using an air conditioner and increasing patient awareness of monitoring local pollen counts in order to avoid unnecessary contact [7].

Strategies to reduce exposure to furry animals include removing the pet from the home, although this recommendation is understandably challenging to follow for most families [40]. Limiting pet access to areas where less allergen exposure is desired (e.g., bedroom) is helpful, as well as washing the pets weekly [40]. Removing reservoirs, such as carpets, is also recommended.

House dust mite control measures include keeping the humidity between 35 and 50%, using mite-allergen proof covers for the bedding, washing the bedding weekly and regular vacuuming with systems using HEPA (high-efficiency particulate air) filters, or with a central vacuum with adequate filtration or that vents to the outside [41]. There is controversy regarding the temperature at which the bedding should be washed for optimal removal of antigens, as mites are likely removed through a combination of drowning and scalding. Certain societies, for example the British Society of Allergy and Clinical Immunology (BSACI), recommend a minimal temperature of 60 °C as this temperature has been shown to kill mite eggs most efficiently [42, 43]. Other bodies such as the American Academy of Allergy, Asthma and Immunology (AAAAI) do not recommend a specific cut-off because high temperature water poses a scalding hazard [41]. Overall, experts agree that washing the bedding weekly helps decrease the antigen burden compared to no washing [44].

One study evaluating the use of an overnight HEPA filter to decrease symptoms in a bedroom environment where Der p 1 and Der f 1 were predominant found a decrease in rhinitis, but the small decrease in eye symptoms noted was not statistically significant [45]. Acaricides for dust mites are discouraged due to their limited efficacy and the concern of harmful chemical exposure [41].

#### Other non-pharmacological measures

Applying cold compresses can alleviate itching by causing conjunctival vasoconstriction, and thereby reducing hyperemia and edema [29]. Lubricant eye drops help to dilute and flush the allergens and inflammatory cells from the tear film, as well as to treat any co-morbid dry eye disease [7]. Wearing large wraparound sunglasses can be used to reduce contact with aeroallergens and improve photophobia [29]. Non-pharmacological measures are variably helpful, have little evidence of efficacy and in most cases are inadequate to control symptoms and signs of AC.

#### Topical dual-activity agents (antihistamine/mast-cell stabilizing activity)

Compared with either antihistamines or mast cell stabilizers, topical dual-activity agents are generally clinically superior due to both symptom/sign relief and tolerability [46]. These are now considered first-line treatment in AC and are the most common ophthalmic agents prescribed by allergists and eyecare practitioners [28]. These agents provide the benefits of two classes of drugs: the immediate relief of antihistamines with the prophylactic benefit of mast cell stabilizers, and as well some have been shown to have other actions including inhibition of eosinophil migration and other mediators of inflammation (e.g. IL-5, PAF, LTB4) [47]. These are used to ameliorate symptoms but may be augmented by other treatments (e.g. steroids) when the signs are also significant or if the presentation is more than just mild.

Dual-activity agents have been well studied and are supported by extensive clinical experience. Examples of topical dual-activity agents include ketotifen 0.025% (Zaditor^®^, Novartis), olopatadine 0.1% (Patanol^®^, Novartis), 0.2% (Pataday^®^, Novartis) and 0.7% (Pazeo^®^, Novartis), as well as bepotastine besilate 1.5% (Bepreve^®^, Bausch & Lomb) (Table 5). Other agents are available in the U.S. but are not yet available in Canada, such as epinastine, alcaftadine and azelastine. All of these agents are preserved with benzalkonium chloride, a surfactant preservative which may cause ocular surface toxicity [48]. When used in those patients who wear contact lenses, drops should be administered at least 15 min prior to lens insertion or after lenses are removed.

Olopatadine was first released in the late 1990s and it has been re-released in various forms since. Compared with placebo, olopatadine has been found to reduce itching and redness, as well as decrease the tear histamine level [49, 50]. Olopatadine has also been shown to decrease chemosis, eyelid edema and significantly improve quality of life [51, 52]. Olopatadine 0.1% was found to be more effective at relieving itching and redness compared to nedocromil sodium 2% in one RCT [53]. In another, ketotifen 0.025% was superior to both placebo and the antihistamine levocabastine 0.05% in relieving itching and watering [54].

Multiple RCTs have compared olopatadine 0.1% with ketotifen 0.025% [55–57]. One metanalysis found improvement in itching at 14 days in favor of olopatadine over ketotifen and no difference in reduction of tearing at 14 days, while another found no difference in efficacy between the two for itch and hyperemia [58, 59].

Bepotastine is the newest available dual-activity agent in Canada and differs in its improved bioavailability, H1 histamine receptor affinity, anti-inflammatory effects as well as rapid onset of action. In two RCTs compared to placebo, bepotastine was found to reduce itching significantly at 15 min with lasting benefit for 8 h after a conjunctival allergen challenge, highlighting both the acute and prolonged effects of the drug [60, 61].

A small cross-over study comparing bepotastine besilate 1.5% with olopatadine 0.2%, both used twice per day, found bepotastine besilate to be more effective for the relief of nasal running/itching and ocular symptoms at both morning and evening time points [62]. Comfort was rated equally and adverse effects were generally mild, though a mild adverse taste was noted in 10% of those using bepotastine besilate. Significantly more subjects preferred bepotastine besilate (63.3%) over olopatadine. Subjects were not masked, however.

#### Steroids: topical ophthalmic and nasal

Steroids treat AC by reducing inflammatory cytokine production, mast cell proliferation and cell mediated immune responses. While very effective, steroids are commonly used for short-term treatment only due to the risk of cataract development and elevated intra-ocular pressure (IOP).

Ophthalmic steroids are often prescribed along with dual-activity agents in the clinical situation where there are both symptoms and noticeable signs, or when the presentation is significant. They may also be used short-term to manage exacerbations or anticipation of periods when exposures to allergens are expected to increase. The ester-based steroid, loteprednol etabonate (0.2% Alrex^®^, 0.5% Lotemax^®^ suspension, gel, both Bausch & Lomb), is the preferred agent for AC. This steroid is metabolized more efficiently therefore reducing the risk of adverse side effects [63]. The 0.2% concentration of loteprednol etabonate is indicated for the treatment of seasonal AC. Only 1% of patients showed a significant IOP rise of ≥ 10 mmHg with this concentration, and its long-term use did not correlate with cataract development [63–65].

Potent ketone-based topical steroids such as prednisolone acetate 1% (Pred Forte^®^, Allergan), prednisolone phosphate 1%, and dexamethasone 0.1% can be prescribed for severe cases of AC. However, these more potent steroids carry more risk of ocular adverse effects and are generally not necessary.

Intranasal steroids used for allergic rhinitis including fluticasone furoate and mometasone furoate have also been shown to have positive effects on ocular allergic symptoms relative to placebo [66, 67]. In one study fluticasone provided superior ocular symptom relief when compared to the oral antihistamine oral fexofenadine [67]. The mechanism of relief may be reduction in the nasal-ocular reflex where the afferent portion is the nasal allergic response and the efferent portion leads to ocular symptoms [68]. This class of medication is therefore often used as first line treatment in allergic rhinoconjunctivitis.

One double blind longitudinal study of 360 patients showed the nasal steroids fluticasone propionate, mometasone furoate, and beclomethasone dipropionate did not cause variations of IOP outside normal limits. However, due to the risk of IOP rise with any steroid use, careful monitoring by applanation tonometry in patients on intranasal steroids is advised [69]. Nasal steroids in addition to topical treatments may be considered when oral antihistamine use causes exacerbation of concomitant dry eye disease due to excessive ocular surface drying.

#### Antihistamines: topical and oral

Oral antihistamines are central in the treatment of allergies. They are easily accessible by patients, both OTC and by prescription.

Oral first-generation antihistamines are best avoided due to their anticholinergic properties and ability to cross the blood–brain barrier [70]. These agents commonly produce undesirable side effects such as confusion, sedation, urinary retention, and dry eyes and mouth, creating the potential to exacerbate any concomitant dry eye disease [71]. Concern also exists related to a possible increased risk of dementia in patients taking high dose anticholinergic medications for a prolonged period of time, as well as an increased risk of falls and fractures in the elderly [72, 73]. Second-generation antihistamines do not cross the blood–brain barrier as readily and produce less anticholinergic effects and are therefore preferred over first-generation antihistamines.

Compared with oral antihistamines, topical antihistamines agents target the ocular tissues directly and have a faster onset of action (3–15 min), a better safety profile and are generally better tolerated due to less systemic absorption [71]. These agents relieve itching and erythema for a short period of time only, necessitating repeated instillations of up to four times per day. Moreover, topical antihistamines have no effect on other mediators of the allergic response like leukotrienes and prostaglandins. Therefore, they are best used in the acute phase reaction and are rarely sufficient as monotherapy.

Antazoline and pheniramine were amongst the first available topical antihistamines and continue to be available OTC in combination with the vasoconstrictor naphazoline (Table 5). Emedastine (Emadine^®^) is a newer and more potent antihistamine, but it is no longer available in Canada, nor is levocabastine (Livostin^®^). Other antihistamines such as cetirizine eye drops are available in the U.S. only. As a rule, topical antihistamines have been usurped by the topical dual-activity agents.

#### Topical NSAIDs

Anti-inflammatory ophthalmic solutions are not often used in AC, but may be useful when symptoms continue to be inadequately controlled despite the use of dual-activity agents or when the prescription of a steroid is not optimal for a particular patient. By blocking the cyclooxygenase pathway, these agents inhibit production of prostaglandins, one of the newly formed mediators of inflammation in IgE mediated allergic responses. The main benefit of a topical NSAID seems to be the temporary reduction of severe symptoms of discomfort.

Examples of NSAIDs used in ocular allergies are ketorolac tromethamine 0.4% (Acular LS^®^, Allergan) and 0.5% (Acular^®^, Allergan), diclofenac sodium 0.1% (Voltaren Ophtha^®^, Novartis) and nepafenac 0.1% (Nevanac^®^, Novartis; Table 5).

Topical NSAIDs are used primarily in perioperative cataract care and were incidentally found to reduce symptoms of AC [7]. Ophthalmic NSAIDs are approved by Health Canada solely for the treatment of perioperative ocular inflammation in cataract surgery and may be used for the treatment of seasonal AC off-label only [74]. Ketorolac tromethamine 0.5% (Acular^®^, Allergan) was approved by the U.S FDA for the treatment of seasonal AC [75].

Topical NSAIDs are generally used short-term, as an add-on to a dual-activity agent. After 7 or 8 days of four times daily use, topical NSAIDs were found to significantly decrease conjunctival inflammation, ocular itching, swollen eyes, discharge/tearing, foreign body sensation and conjunctival injection [76]. Adverse effects include significant irritation on instillation and rarely keratitis, corneal ulceration or perforation [77].

#### Topical vasoconstrictors

Over-the counter-topical vasoconstrictors are readily available. They can help reduce erythema but have a limited effect on pruritus [46]. Moreover they can cause stinging and burning upon instillation, as well as tachyphylaxis and rebound hyperemia upon discontinuation of use [78]. They are best used as a short-term solution [71–73].

#### Topical mast-cell stabilizers

Topical mast-cell stabilizers inhibit mast cell degranulation by an unclear mechanism of action [18]. Examples of available mast cell stabilizers include lodoxamide (Alomide^®^, Novartis) and sodium cromoglycate 2%, the latter of which is OTC (Table 5). They are best utilized on a prophylactic basis and require a loading period of a few weeks prior to allergen exposure [18]. When used prophylactically, they have been found to reduce itching and tearing compared to placebo in several randomized control trials (RCTs) [79–81]. Due to the availability of more effective therapies in the dual-activity agents, mast cell stabilizers are also seldomly used as monotherapy.

#### Immunotherapy

Immunotherapy is the only therapy that can provide continued benefits after an adequate course is completed. Immunologic changes involve downregulation of the Th2 response and upregulation of regulatory T cells that produce inhibitory cytokines. This ultimately leads to a reduced end-organ response to allergen exposure [82].

Two forms of immunotherapy are approved in Canada: sublingual immunotherapy (SLIT) and subcutaneous immunotherapy (SCIT). SCIT is further divided in pre-seasonal or year-round treatments depending on patient preference and availability of allergens. Other delivery methods for immunotherapy such as intralymphatic IT and local conjunctival IT exist and are not approved for use in Canada at this time.

Both SCIT and SLIT are recommended for the treatment of allergic rhinoconjunctivitis [46, 82, 83].

- Subcutaneous immunotherapy (SCIT): pre-seasonal and year round
  Subcutaneous immunotherapy (SCIT) was introduced by Noon in 1911 as a means to treat symptoms caused by environmental allergies [84]. SCIT is recommended for the treatment of AC and should be continued for 3 to 5 years to induce sustained clinical remission [82].
  Only three allergens (trees, ragweed and grass), are available as pre-seasonal injections. All other common allergens are available for year-round therapy, although only 14 agents treating allergic rhinoconjunctivitis are standardized: cat [2], grass pollen [8], house dust mites [2] and short ragweed. As a side note, the only other standardized allergenic extracts currently available are Hymenoptera venoms [6, 85].
  SCIT benefits from extensive experience and multiple studies confirm its efficacy in the treatment of AC. One systematic review including 11 studies reporting on conjunctival symptoms concludes that there is strong evidence that SCIT to grass mix, timothy grass, cat, Parietaria and Alternaria improves symptoms of conjunctivitis [86]. Another systematic review including 3 studies reporting on conjunctival symptoms also supports SCIT to Parietaria and grass mix as treatment of AC [87].
  One small trial compared pre-seasonal IT to perennial IT for symptom reduction using Allergovit^®^ (Allergopharma; 80% grass pollen, 20% rye pollen). Both the pre-seasonal and perennial groups received 7 injections every 1 to 2 weeks up to a dose of 0.6 mL of a 10,000 therapeutic units/mL concentration (hence a final dose of 4800 TU of grass pollen and 1200 TU of rye pollen per injection). The pre-seasonal group continued with histamine placebo injections every 4 to 6 weeks and the perennial group continued with 0.6 mL of the extract at the same time interval. After 3 years of treatment, both groups had significant reduction in ocular symptoms compared to baseline but there was no significant difference between the two treatment arms. Total rhinoconjunctivitis symptoms and use of rescue medication were lower in the perennial immunotherapy group [88].
- Sublingual immunotherapy (SLIT)
  Sublingual immunotherapy is the newest form of immunotherapy available and can be delivered both as dissolvable tablets or extract solution. Only tablets are available in Canada: Oralair^®^ (Stallergenes Greer; for the treatment of grass pollen allergy) became available in 2012, Grastek^®^ (ALK; grass) and Ragwitek^®^ (ALK; short ragweed) in 2014 and the newest therapy, Acarizax^®^ (ALK; house dust mites), was introduced in 2017. This review will focus on tablets given their availability in Canada.
  Early reports on the efficacy of SLIT contained information related to rhinitis, while effects on conjunctivitis were explored later. A recent meta-analysis looking at the use of SLIT in AC included 13 RCTs and 1592 patients aged 3–18 and evaluated response to olive pollen, parietaria pollen, house dust mites and grass pollen mix immunotherapy [89]. The studies used either sublingual tablets or drops. All RCTs reported on allergic rhinoconjunctivitis and none solely on AC.
  Treatment of pollen-induced AC with SLIT was significantly effective in improving total ocular symptom scores and reducing ocular redness, itch and tearing, while treatment of house dust mite-induced AC was not. There was a trend towards a lower efficacy of drops compared to tablets, although no RCTs compared to two head-to-head [89]. Two RCTs included other medication use and showed no decrease in the placebo versus SLIT groups. The combined drop-out rate of all patients on SLIT was 10.1%.
  Another meta-analysis looking at combined pediatric and adult populations included 42 RCTs with 3958 patients of a median age of 29.7 and evaluated response to grass pollen, tree pollen, house dust mites, weeds and cat extract immunotherapy [90]. The studies used either drops, tablets or both drops during the build-up phase and tablets for the maintenance phase. There was significant reduction in total ocular symptom scores and in ocular signs (redness, itch and tearing) compared to placebo in pollen-induced AC, but not to house dust mites in the pediatric population. Once again, there was no reduction in ophthalmic medication use [90].

#### Biologics

Omalizumab is a humanized monoclonal antibody that binds to the FCεR3 portion of unbound IgE. Two RCTs have compared omalizumab with placebo and report the effects of the drug on AC [91, 92]. They show significant reduction in nasal and ocular symptoms (red, itchy, watery eyes) in the omalizumab group compared with placebo after 12 and 16 weeks. Omalizumab has not been studied in the treatment of AC outside of research done on allergic rhinitis. Case reports exist showing good effect of omalizumab for treatment of atopic keratoconjunctivitis (AKC) and vernal keratoconjunctivitis (VKC) [93–95]. Omalizumab is not approved for treatment of allergic eye disease.

Dupilumab (an IL-4 and IL-13 pathway inhibitor) has not been studied in AC, but one reported adverse effect of the drug is conjunctivitis, described as inflammation of the anterior conjunctiva and hyperemia of the limbus [96]. Incidence varies from 5 to 28% in dupilumab groups, compared with 2–11% in placebo groups [97–99]. Pre-existing AC appears to be a risk factor and dupilumab-related conjunctivitis seems to respond to fluorometholone 0.1% eye drops or off-label tacrolimus 0.03% eye ointment [96].

Neither mepolizumab, reslizumab or benralizumab (anti IL-5 biologic agents) have been studied in the context of AC.

#### Future directions for topical treatments

As discussed above, topical steroids are successful in treating AC. With the ester-based steroids, there is less risk of adverse effects including IOP elevation and cataract formation. Mapracorat is a selective glucocorticoid receptor agonist that is non-steroidal and is currently in clinical trials for ocular use. Mapracorat diminishes recruitment of eosinophils and inflammation inducing cytokine production in experimental ocular models. Encouragingly, Mapracorat raises IOP less than the topical steroid dexamethasone in these models [100].

Cyclosporine A reduces the allergic response by suppressing T lymphocyte proliferation and inflammatory cytokine activity thereby inhibiting histamine release from mast cells and basophils and reducing eosinophil recruitment [101]. A systematic review suggested topical cyclosporine could be used to treat AC and help reduce the reliance on topical steroids (and therefore risk of IOP increase and cataracts) in more severe cases. Topical cyclosporine was found to be safe with the major side effects being burning and stinging on instillation. For different types of AC and severities of patient presentations, more studies are needed to provide information on the appropriate concentration of cyclosporine (various concentrations from 0.05 to 2% have been used in studies). It is important to note that Health Canada has not authorized the use of cyclosporine ophthalmic emulsion 0.05% in patients under the age of 18 as there is not yet enough data on its safety in the pediatric population [102, 103].

Topical calcineurin inhibitors such as tacrolimus and pimecrolimus are effective treatments for atopic dermatitis; however, they are not yet available for ophthalmic use. A study in Japan using tacrolimus 0.1% drops showed promise in treating AC (including cases that were unresponsive to topical cyclosporine). The possible link between long-term tacrolimus use and risk of malignancy underlies the need for more research.

### Proposed pharmacologic treatment algorithm for the management of allergic conjunctivitis

**Fig. 5:**
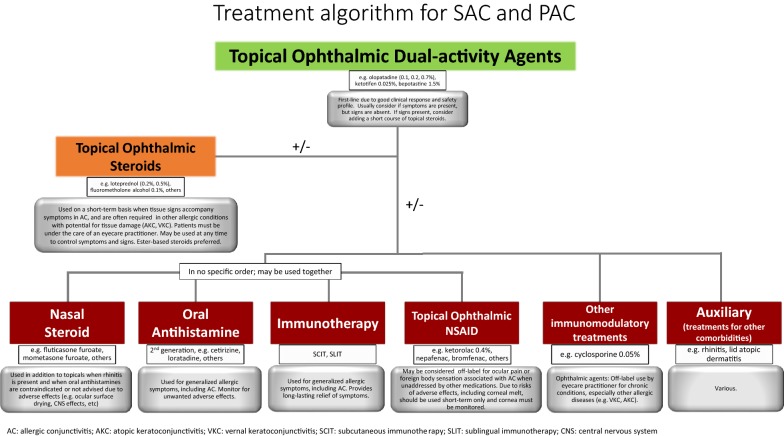
Treatment strategies for the management of allergic conjunctivitis

**Fig. 6:**
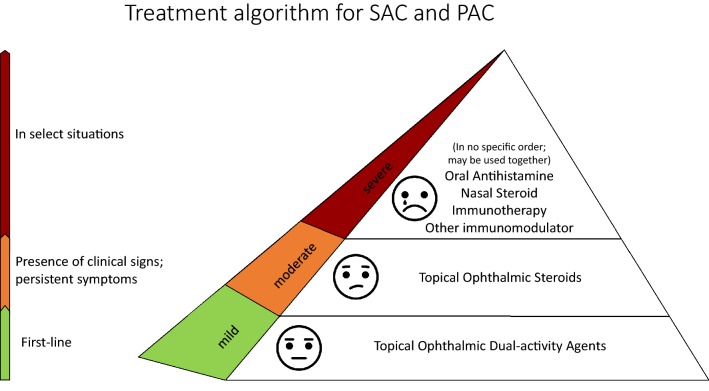
A simplified approach to the treatment of allergic conjunctivitis

The pharmacologic treatment of AC is focused on relief of symptoms and resolution of signs, if present. We propose the following treatment algorithms based on expert opinion. The first algorithm (Fig. 5) presents a more extensive overview to the management of AC, whereas the second algorithm (Fig. 6) provides a simplified overview.

As a first step, the diagnosis of AC should be confirmed and the severity assessed. Determining the severity, both in terms of impact on the patient’s QoL and physical examination findings, can help clinicians choose the appropriate strategies to provide prompt and maximal relief.

The dual-activity agents are considered the appropriate first-line therapy. They are easily accessible and well tolerated. When symptoms and signs remain uncontrolled, a short course of topical ophthalmic steroids may be considered. As discussed above, monitoring by an eye care specialist is necessary when there is consideration to use this agent on a longer term basis. Other treatments include nasal steroids, oral anti-histamines and/or topical ophthalmic NSAIDs, which are listed in no particular order and can be used concomitantly. Topical calcineurin inhibitors can be used off-label by eyecare specialists as a next step. Immunotherapy, either subcutaneous or sublingual, can provide a longer term solution to the symptoms and signs of AC and can be considered when medical therapy is insufficient, poorly tolerated or for patient preference.

### Interprofessional collaboration

**Fig. 7:**
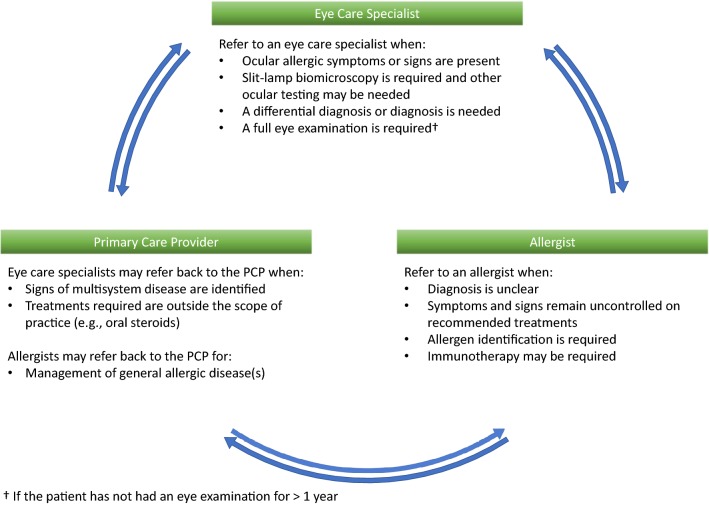
Interprofessional collaboration—conditions for patient referral to an eye care specialist, a primary care provider (PCP) and an allergist [15]

Allergic eye disease is increasingly being recognized amongst health care professionals. Patients may initially consult various practitioners and a multidisciplinary approach is crucial in ensuring adequate diagnosis, counselling and treatment. The primary care provider, optometrist, ophthalmologist and allergist are key players in patient care. Figure 7 illustrates conditions where evaluation by various specialists is recommended.

A thorough eye examination is difficult to complete in a primary care provider or allergist’s office, given the limitations of equipment availability. Referral to an optometrist or ophthalmologist should be considered when a full eye examination has not been done in the past year, as they can perform proper slit-lamp biomicroscopy. Eye care specialists can be especially helpful if symptoms are not typical of uncomplicated AC, pain is present, the diagnosis is unclear, symptoms persist despite adequate therapy, signs are present suggesting the need for more than a dual-activity agent and/or to monitor response to therapy and ophthalmic adverse effects, including but not limited to steroids [15].

An allergist is most helpful when symptoms remain uncontrolled after empiric therapy, sensitization needs to be determined (skin prick testing or serum specific IgE) or immunotherapy is indicated.

The allergist and eye care specialist may refer back to the primary care provider when signs and symptoms are well controlled and require chronic management. Communication between all practitioners involved is essential for optimal care.

## Conclusion

AC and other ocular allergic diseases are highly prevalent yet continue to remain underdiagnosed and undertreated. Signs and symptoms of AC can significantly impair quality of life. A thorough history and physical examination is key in identifying AC and ruling out other diagnoses. A multitude of pharmacological options are available and the choice of therapy should be tailored to each patient. Primary care providers, eye care specialists and allergists each play an important role in patient management and a multidisciplinary approach is essential to maximize care.

## Data Availability

Not applicable.

## Abbreviations

AC: allergic conjunctivitis
QoL: quality of life
NSAIDs: non-steroidal anti-inflammatory drugs
AKC: atopic keratoconjunctivitis
VKC: vernal keratoconjunctivitis
CLPC: contact-lens associated papillary conjunctivitis
GPC: giant papillary conjunctivitis
IgE: immunoglobulin E
IL: interleukin
IT: immunotherapy
HEPA: high-efficiency particulate air
SCIT: subcutaneous immunotherapy
SLIT: sublingual immunotherapy
IOP: intra-ocular pressure
RCT: randomized controlled trial
OTC: over-the-counter
BID: twice daily
TID: three times a day
QID: four times a day
N/A: not available
Rx: prescription
PCP: primary care provider
ADHD: attention deficit hyperactivity disorder
